# Farmers’ choice of genotypes and trait preferences in tropically adapted chickens in five agro-ecological zones in Nigeria

**DOI:** 10.1007/s11250-019-01993-0

**Published:** 2019-07-15

**Authors:** A. Yakubu, O. Bamidele, W. A. Hassan, F. O. Ajayi, U. E. Ogundu, O. Alabi, E. B. Sonaiya, O. A. Adebambo

**Affiliations:** 1 Department of Animal Science, Faculty of Agriculture, Nasarawa State University, Keffi, Shabu-Lafia Campus, Lafia, Nasarawa Nigeria; 2 African Chicken Genetic Gains (ACGG) Project National Secretariat, Department of Animal Science, Obafemi Awolowo University, Ile-Ife, Osun Nigeria; 3 Department of Animal Science, Usmanu Danfodiyo University, Sokoto, Sokoto Nigeria; 4 Department of Animal Science, University of Port-Harcourt, Rivers State, Nigeria; 5 Department of Animal Science, Federal University of Technology, Owerri, Imo Nigeria; 6 Department of Animal Science, Landmark University, Omu-Aran, Kwara Nigeria; 7 Department of Animal Breeding and Genetics, Federal University of Agriculture, Abeokuta, Ogun Nigeria

**Keywords:** Chicken, Traits, Non-parametric, Multivariate analysis, Tropics

## Abstract

This study aimed at determining chicken genotypes of choice and traits preference in chicken by smallholder farmers in Nigeria. Data were obtained from a total of 2063 farmers using structured questionnaires in five agro-ecological zones in Nigeria. Chi square (*χ*^2^) statistics was used to explore relationships between categorical variables. The mean ranks of the six genotypes and twelve traits of preference were compared using the non-parametric Kruskal–Wallis *H* (with Mann–Whitney *U* test for post hoc separation of mean ranks), Friedman, and Wilcoxon signed-rank (with Bonferroni’s adjustments) tests. Categorical principal component analysis (CATPCA) was used to assign farmers into groups. Gender distribution of farmers was found to be statistically significant (*χ*^2^ = 16.599; *P* ≤ 0.002) across the zones. With the exception of Shika Brown, preferences for chicken genotypes were significantly (*P* ≤ 0.01) influenced by agro-ecological zone. However, gender differentiated response was only significant (*P* ≤ 0.01) in Sasso chicken with more preference by male farmers. Overall, FUNAAB Alpha, Sasso, and Noiler chicken were ranked 1st, followed by Kuroiler (4th), Shika Brown (5th), and Fulani birds (6th), respectively. Within genotypes, within and across zones and gender, preferences for traits varied significantly (*P* ≤ 0.005 and *P* ≤ 0.01). Traits of preference for selection of chicken breeding stock tended towards body size, egg number, egg size, and meat taste. Spearman’s rank order correlation coefficients of traits of preference were significant (*P* ≤ 0.01) and ranged from 0.22 to 0.90. The two PCs extracted, which explained 65.3% of the variability in the dataset, were able to assign the farmers into two groups based on preference for body size of cock and hen and the other ten traits combined. The present findings may guide the choice of appropriate chicken genotypes while the traits of economic importance may be incorporated into future genetic improvement and conservation programs in Nigeria.

## Introduction

Indigenous chicken are widely distributed in the rural areas of tropical and sub-tropical countries (Ajayi 2010). The birds play a key role for the poor farmers and the underprivileged within the rural setting as regards subsidiary income, provision of chicken meat and eggs (Padhi 2016) and food security (Melesse 2014). In spite of this, smallholder poultry sub-sector in sub-Saharan Africa is beset with myriad of problems among which are poor nutrition, limited technical know-how, vagaries of climatic factors, slow-growing, low meat yield, small size/number of eggs, low input, and high mortality (Yakubu, 2010; Ayanwale et al. 2015; Dessie, 2017).

In order to address the factors militating against high chicken production and productivity at the smallholder level, research efforts in the area of genetics and breeding “among others” have been made in the past three decades (Adedokun and Sonaiya 2002; Sonaiya 2016). One of such is the development of chicken genotypes that are adapted to the prevailing tropical conditions (Adebambo et al. 2018). However, it has been reported that the proper identification of appropriate chicken breeds that will be suitable to a particular environment or agro-ecological zone in Nigeria is required for the growth and development of the poultry industry (Hassan et al. 2018). Such decision on the chicken genotypes of preference is expected to be based on farmers’ choice especially at the smallholder level using the bottom-top approach. This coupled with farmers’ traits of preference may be valuable inputs for appropriate design and implementation of agro-ecologically friendly and sustainable genetic improvement programs of the indigenous stock. Knowledge of trait preferences for breeding decisions is central to the formulation of livestock policies aimed at improving the livelihoods of smallholder chicken farmers. Evaluation of trait preferences of local poultry producers is required for the design of appropriate breeding programs (Brown et al. 2017). This may be particularly indispensable under the free scavenging production system (Markos et al. 2016), where the economic weights of traits could be difficult to calculate and also permit the inclusion of non-market traits in the economic valuation of the chicken (Bett et al. 2011). This assertion is believed to be workable only when due emphasis has been laid on the phenotypic and genetic correlations as well as the heritability of the traits. This is in consonance with the recommendations of Woldu et al. (2016), Traoré et al. (2017), and Perucho et al. (2019). The attendant effect may be holistic improvement, sustainable utilization, as well as rational conservation of the indigenous chicken to improve the living standard of the smallholder farmers (Markos et al. 2016). However, future breeding studies on preference traits should also put into consideration the interests of marketers and consumers. This is because of the probable rejection of chicken/chicken products that do not include the traits of preference of some critical stakeholders along the poultry value chain. Similar findings were reported by Okeno et al. (2011) where breeding programs designed without inputs from all the relevant stakeholders stood a high risk of being rejected by the end users.

Under the African Chicken Genetic Gains (ACGG) project, Kuroiler and Sasso birds (foreign, but tropically adapted genotypes) alongside the newly developed Nigerian indigenous FUNAAB alpha, as well as the Shika Brown, Fulani, and Noiler chicken were tested in five agroecological zones of Nigeria. This study, therefore, aimed at evaluating choice of chicken genotypes and trait preferences by smallholder chicken farmers in Nigeria. This may assist in future research efforts on genotypes and traits of economic importance by private and public intervention programs geared towards boosting smallholder chicken production.

## Materials and methods

### Description of study area

The post on-farm data collection study was conducted in five agro-ecological zones under the African Chicken Genetic Gains (ACGG) project in Nigeria. The ACGG is a platform for testing, delivering, and continuously improving tropically adapted chickens for productivity growth in 3 selected African countries: Ethiopia, Tanzania, and Nigeria (www.africacgg.net). In Nigeria, the on-farm test was conducted from 2016 to 2018. It was a randomized complete block design (RCBD) of 420 farmers per agro-ecology. The breeds were randomly allocated to the farmers, and each farmer received one breed of 30 birds at 6 weeks old. The birds were managed under the traditional poultry scavenging system in all the five zones. Each zone was represented by a State [Kwara (Humid Kishi-Ilorin-Kabba Plain), Rivers (Very Humid/Per Humid Niger-Delta), Imo (Very Humid Onitsha-Enugu-Abakaliki-Calabar Lowland and Scarpland), Nasarawa (Sub-Humid Central Niger-Benue Trough) and Kebbi (Dry Sub-Humid Illela-Sokoto-Yelwa Plain)] as delineated by NSPFS (2005) (Table 1).

**Table 1:** Main features and differences between the agro-ecological zones

Features	Zone				
	Kwara	Rivers	Imo	Nasarawa	Kebbi
GPS coordinates	Between latitudes 8° 30′ N and 8° 50′ N and longitudes 4° E 20′ and 4° 35′ E	Latitude 4° 45**′** N and longitude 6° 50′ E	Between latitudes 4^°^ 45′ N and 7^°^ 15′ N and longitudes 6^°^ 50′ E and 7^°^ 25′ E	Between latitudes 7^°^ 52′ N and 8^°^ 56′ N and longitudes 7^°^ 25′ E and 9^°^ 37′ E	Latitude 4° 45**′** N and longitude 6° 50′ E
Temperature (°C)	26.8	26.7	26.4	28.4	28.4
Relative humidity (%)	74.4	83.4	80.0	74.0	47.4
Rainfall (mm, per annum)	1217	2708	2219	1169	807
Land mass (km^2^)	35,705	10,575	5,288	28,735	36,985
Human population	2,365,353	5,198,716	3,927,563	1,869,377	3,256, 541
Major ethnic group	Yoruba	Ogoni	Igbo	Eggon	Hausa-Fulani
Major economic activities	Agriculture	Oil, agriculture, and fishing	Agriculture and oil	Agriculture and solid minerals	Agriculture and fishing

Sources: NPC (2006); NBS (2011); Eludoyin et al. (2013); Esiobu and Onubuogu (2014)

### Management of birds

The feeding of birds was supplemented with readily available commercial feeds, agricultural by-products and kitchen wastes. Health management practice was also carried out based on the capacity of the farmers. The study was conducted between December 2017 and August 2018.

### Sampling procedure

A total of 2100 (420 per zone) rural chicken keepers from five zones (Kwara, Rivers, Imo, Nasarawa, and Kebbi) were randomly sampled. In each zone, twelve villages, two per local government area (LGA) in each of the three senatorial districts were randomly selected. However, data for final analysis were only available for 2063 farmers. The distribution of the participating farmers that were earlier given a certain number of Sasso, Kuroiler, Fulani, Shika Brown, Noiler, and FUNAAB alpha birds for the on-farm testing (for periodic performance data collection such as body weight and egg parameters of birds) is shown in Table 2. The ethical guidelines of International Livestock Research Institute (ILRI), Ethiopia, were strictly adhered to. The present study was approved by ILRI Institutional Research Ethics Committee (ILRI IREC) with reference no.: ILRI-IREC2015-08/1. Each farmer also gave written informed consent to participate in the study in line with best global practices.

**Table 2:** The distribution of respondents based on zone and chicken genotype

		Genotype					
Zone	Fulani	FUNAAB Alpha	Shika Brown	Noiler	Kuroiler	Sasso	Total
Kwara	36	48	83	84	84	84	419
Rivers	33	44	77	77	77	77	385
Imo	36	48	84	84	84	84	420
Nasarawa	36	48	84	84	84	84	420
Kebbi	36	48	84	84	84	83	419
Grand total							2063

### Data collection procedure

Structured questionnaires were used to elicit information on the gender of farmers, the choice of chicken genotypes and traits of preference in a post on-farm data collection survey. During the on-farm test, the farmers met every quarter in each project village, at the community innovation platform, to among other things compare the performance of the breeds allocated to them. Based on individual experience over time, each farmer was asked to assess subjectively the performance of the genotype given to him/her and indicate Yes/No his or her preference for the genotype. Where the response was not in the affirmative, the farmer was asked to indicate a ready alternative chicken genotype to the one he/she was given. Information on the farmers’ preferences for traits of economic importance that influenced their choice of a particular genotype was also obtained. The traits (body size–cock; body size–hen; supplementary feed consumption–cock; supplementary feed consumption–hen; egg number–hen; egg size–hen; scavenging ability–cock; scavenging ability–hen; meat taste–cock; meat taste–hen; ease of sales–cock and ease of sales–hen) as perceived by the respondents were ranked on a scale of one (Like very much), two (Like), three (Not Important), four (Dislike), five (Dislike very much), six (Not Applicable).

### Statistical analysis

Chi square (*χ*^2^) statistics was used to explore relationships between the gender of farmers and zones; chicken genotype of choice by the farmers as well as the alternative genotype across zones and according to gender. The non-parametric Kruskal–Wallis *H* one-way analysis-of-variance test followed by the Mann–Whitney *U* test for post hoc separation was used to compare mean ranks of the five genotypes in order of preference by farmers. Means and their standard deviation of rankings were also calculated for within-genotype comparison, within- and between-zone comparisons and within-and between-gender comparisons of the traits of economic importance. Within each genotype, zone and gender, comparisons of means were performed using the Friedman test: This test compares the distribution of preference ranks of each trait of economic importance. Post hoc analyses were then applied using the non-parametric Wilcoxon signed-rank test with Bonferroni’s adjustments (Dossa et al. 2015; Yakubu et al. 2019). The non-parametric Kruskal–Wallis *H* test followed by the Mann–Whitney *U* test for post hoc separation of mean ranks of the traits of economic importance was also used for the comparison between zones and gender.

In order to explore hidden patterns of trait preferences for appropriate grouping of the respondents, categorical principal component analysis (CATPCA) procedure was used as described by Martin-Collado et al. (2015). The varimax criterion with Kaiser normalization was used to rotate the PC matrix to facilitate easy interpretation of the analysis. Chronbach’s alpha was used to test the reliability of the PCA. The PCA was preceded by Spearman’s rank order correlation analysis of farmers’ traits of preference to indicate the directional effects and plausible trade-offs between traits. IBM (2015) statistical package was employed in the analysis.

## Resu**l**ts

**Fig. 1:**
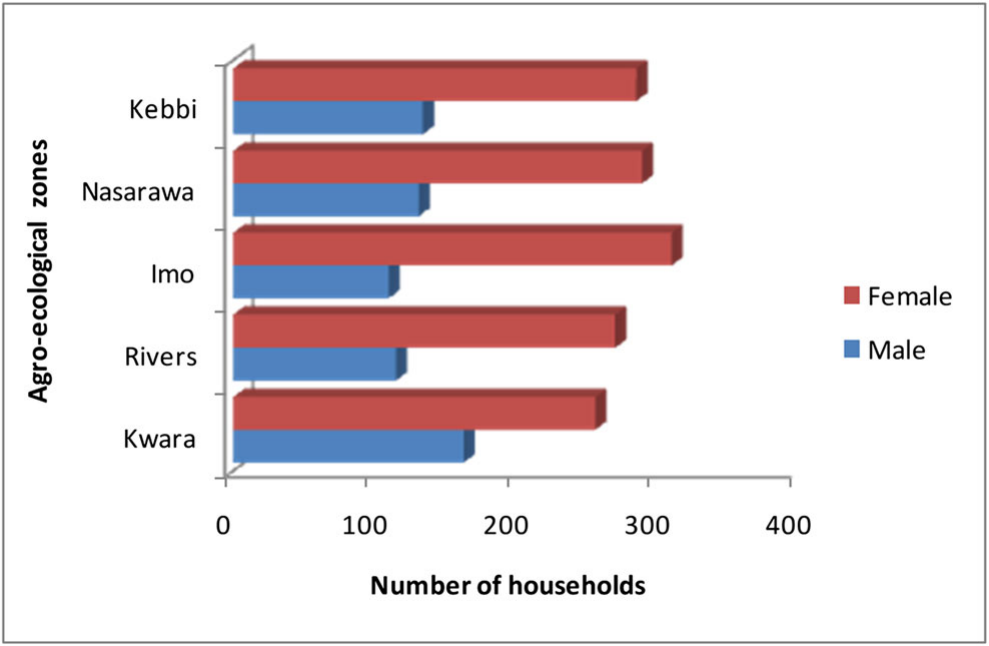
Gender distribution of households

Gender distribution was found to be statistically significant (*χ*^2^ = 16.599; *P* ≤ 0.002) across the zones. More male households were found in Kwara (163, 38.9%), Kebbi (134, 32.0%), and Nasarawa (131, 31.2%) while the female respondents were more in Imo (310, 73.8%), Nasarawa (289, 68.8%), and Kebbi (285, 68.0%), respectively (Fig. 1).

The preference for a chicken genotype was significantly (*P* ≤ 0.01) influenced by agro-ecological zone with the exception of Shika Brown (percentage likeness for this genotype ranged from 52.6 to 72.3%) (Table 3). There was high preference for FUNAAB Alpha in Rivers (90.9%), Nasarawa (89.6%), and Kebbi (87.5%), respectively. The preference for Kuroiler was also high in Imo (88.1%), Rivers (83.1%), and Kwara (81.0%). Similarly, 91.7 (Imo), 88.0 (Rivers), 79.8 (Nasarawa), and 73.8% (Kwara) of the farmers given Sasso chicken expressed their likeness for the birds. On the other hand, 88.1 (Nasarawa), 86.9 (Imo), and 79.8 (both Kwara and Kebbi) showed high preference for Noiler birds. However, the Fulani birds were least preferred by farmers across zones (5.6–48.5%).

**Table 3:** Chicken genotype preference by farmers across zones in Nigeria

	Zone						
	Kwara	Rivers	Imo	Nasarawa	Kebbi		
Factor	No. (%)	No. (%)	No. (%)	No. (%)	No. (%)	Chi-square	*P* value
Genotype							
Shika Brown							
Liked	49 (59.0)	40 (52.6)	60 (72.3)	48 (57.1)	52 (61.9)		
Not liked	34 (41.0)	36 (47.4)	23 (27.7)	36 (42.9)	32 (38.1)	7.342	0.119^ns^
FUNAAB Alpha							
Liked	38 (79.2)	40 (90.9)	30 (62.5)	43 (89.6)	42 (87.5)		
Not liked	10 (20.8)	4 (9.1)	18 (37.5)	5 (10.4)	6 (12.5)	17.671	0.01**
Fulani							
Liked	17 (47.2)	16 (48.5)	3 (8.3)	2 (5.6)	14 (38.9)		
Not liked	19 (52.8)	17 (51.5)	33 (91.7)	34 (94.4)	22 (61.1)	30.433	0.01**
Kuroiler							
Liked	68 (81.0)	64 (83.1)	74 (88.1)	58 (69.0)	52 (64.2)		
Not liked	16 (19.0)	13 (16.9)	10 (11.9)	26 (31.0)	29 (35.8)	18.743	0.01**
Sasso							
Liked	62 (73.8)	66 (88.0)	77 (91.7)	67 (79.8)	50 (60.2)		
Not liked	22 (26.2)	9 (12.0)	7 (8.3)	17 (20.2)	33 (39.8)	30.246	0.01**
Noiler							
Liked	67 (79.8)	47 (61.0)	73 (86.9)	74 (88.1)	67 (79.8)		
Not liked	17 (20.2)	30 (39.0)	11 (13.1)	10 (11.9)	17 (20.2)	22.675	0.01**

**^ns^Significant at *P* ≤ 0.01; not significant

The likeness of Shika Brown, FUNAAB Alpha, Fulani, Kuroiler, and Noiler birds was not significantly (*P* > 0.05) influenced by gender (Table 4). However, there was significant (*P* ≤ 0.05) gender effect as regards preference for Sasso chicken in the direction of male farmers.

**Table 4:** Chicken genotype preference according to gender of farmers in Nigeria

	Gender			
	Male	Female		
Factor	No. (%)	No. (%)	Chi-square	*P* value
Genotype				
Shika Brown				
Liked	71 (58.2)	178 (61.8)		
Not liked	51 (41.8)	110 (38.2)	0.468	0.494^ns^
FUNAAB Alpha				
Liked	62 (81.6)	131 (81.9)		
Not liked	14 (18.4)	29 (18.1)	0.003	0.956^ns^
Fulani				
Liked	20 (33.3)	32 (27.4)		
Not liked	40 (66.7)	85 (72.6)	0.684	0.408^ns^
Kuroiler				
Liked	93 (79.5)	223 (76.1)		
Not liked	24 (20.5)	70 (23.9)	0.540	0.462^ns^
Sasso				
Liked	109 (85.8)	213 (75.3)		
Not liked	18 (14.2)	70 (24.7)	0.119	0.016*
Noiler				
Liked	121 (81.8)	207 (78.1)		
Not liked	27 (18.2)	58 (21.9)	0.771	0.380^ns^

*Significant at *P* ≤ 0.05; ^ns^not significant

Out of a total of 599 farmers who did not like the particular genotypes given to them; when they were asked to indicate the alternative genotypes they preferred, their interest varied significantly (chi-square = 230.006; *P* ≤ 0.01) across zones (Table 5). From this, there was high preference for Sasso, Noiler, FUNAAB Alpha, and Kuroiler.

**Table 5:** Alternative chicken genotype preference by farmers across zones in Nigeria

	Zone						
	Kwara	Rivers	Imo	Nasarawa	Kebbi		
Factor	No, (%)	No, (%)	No, (%)	No, (%)	No, (%)	Chi-square	*P* value
Genotype							
Shika Brown	5 (4.3)	9 (8.3)	17 (16.2)	3 (2.3)	14 (10.0)		
FUNAAB α	20 (17.1)	17 (15.6)	5 (4.8)	12 (9.4)	56 (40.0)		
Fulani	11 (9.4)	2 (1.8)	1 (1.0)	0 (0.0)	6 (4.3)		
Kuroiler	32 (27.4)	19 (17.4)	19 (18.1)	13 (10.2)	11 (7.9)		
Sasso	21 (17.9)	56 (51.4)	54 (51.4)	43 (33.6)	6 (4.3)		
Noiler	28 (23.9)	6 (5.5)	9 (8.6)	57 (38.8)	47 (33.6)	230.006	0.01**

*α* alpha

**Significant at *P* ≤ 0.01

**Fig. 2:**
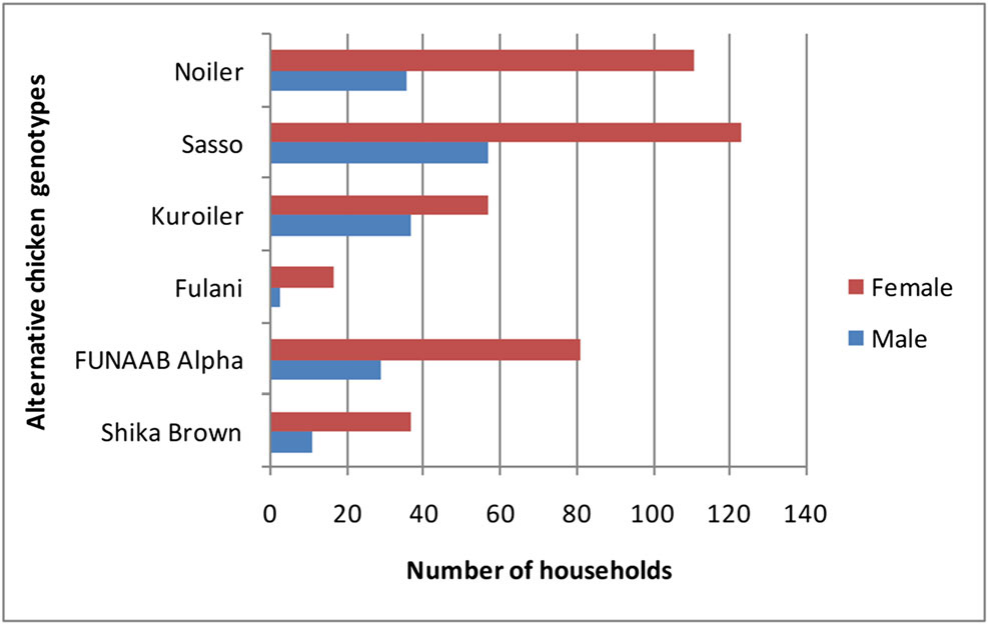
The distribution of the alternative genotypes based on gender

Gender had no significant effect (chi-square = 10.134; *P* ≤ 0.07) across alternative genotypes, although the number of female farmers was higher (Fig. 2): males [Shika Brown (11, 6.4%), FUNAAB Alpha (29, 16.8%), Fulani (3, 1.7%), Kuroiler (37, 21.4%), Sasso (57, 32.9%), and Noiler (36, 20.8%)] and females [Shika Brown (37, 8.7%), FUNAAB Alpha (81, 19.0%), Fulani (17, 4.0%), Kuroiler (57, 13.4%), Sasso (123, 28.9%), and Noiler (111, 26.1%)].

In order to appropriately rank the five chicken genotypes, data on actual chicken genotype preferences across zones (Table 3 above) and those of the alternative genotypes (Table 5 above) were combined (Table 6). Equal ranking (1st position) was observed in the case of FUNAAB Alpha, Sasso, and Noiler birds. Kuroiler, Shika Brown, and Fulani chickens were ranked 4th, 5th, and 6th, respectively.

**Table 6:** Ranking of preferred chicken genotypes by farmers in Nigeria

Genotype	Liked	Not liked	Mean rank^a^	Kruskall–Wallis test	Position
	No. (%)	No. (%)			
Shika Brown	297 (64.8)	161 (35.2)	1496.65c		5th
FUNAAB Alpha	303 (87.6)	43 (12.4)	1194.98a		1st
Fulani	72 (36.5)	125 (63.5)	1872.32d		6th
Kuroiler	410 (81.3)	94 (18.7)	1277.59b		4th
Sasso	502 (85.1)	88 (14. 9)	1228.00ab		1st
Noiler	475 (84.8)	85 (15.2)	1231.50ab	292.970**	1st

Means in columns followed by different letters are different significantly (*P* ≤ 0.05)

**Significant at *P* ≤ 0.01

^a^The lower the mean, the more important the genotype

Within-genotype ranking of the chickens is shown in Table 7. Farmers appeared to attach importance (*P* ≤ 0.01) to BSC, BSH, ENH, EZH, MTC, and MTH in the choice of Shika Brown, FUNAAB Alpha, Fulani, Kuroiler, Sasso, and Noiler chickens (Table 5). Additionally, SAH, SAC, SFH, and SFC were highly (*P* ≤ 0.01) ranked in Fulani birds while Noiler farmers also rated higher SFH and SFC.

**Table 7:** Mean (± SD) of traits preference in six chicken genotypes and their significance level according to Friedman test

	Genotype					
	Shika Brown	FUNAAB Alpha	Fulani	Kuroiler	Sasso	Noiler
Traits	Mean^a^	Mean^a^	Mean^a^	Mean^a^	Mean^a^	Mean^a^
BSC	1.45 ± 0.68a	1.32 ± 0.68a	1.85 ± 0.99a	1.46 ± 0.89a	1.54 ± 1.05a	1.39 ± 0.69a
BSH	1.58 ± 0.83b	1.37 ± 0.67a	1.74 ± 0.92a	1.52 ± 0.89a	1.61 ± 1.07a	1.44 ± 0.74a
SFC	2.09 ± 1.28d	2.05 ± 1.31c	2.02 ± 1.09ab	2.08 ± 1.23c	2.30 ± 1.31d	1.86 ± 1.15b
SFH	2.04 ± 1.18d	1.97 ± 1.17c	1.97 ± 1.07a	2.07 ± 1.21c	2.27 ± 1.19d	1.80 ± 0.95b
ENH	1.66 ± 1.08b	1.72 ± 0.96b	2.03 ± 1.04ab	2.02 ± 1.24c	2.13 ± 1.23c	1.73 ± 0.84b
EZH	1.70 ± 1.14b	1.81 ± 1.03bc	2.44 ± 1.28b	1.90 ± 1.19b	2.05 ± 1.23c	1.75 ± 0.81b
SAC	1.98 ± 1.15cd	2.05 ± 1.37c	1.81 ± 0.90a	2.16 ± 1.32c	2.26 ± 1.32d	2.09 ± 1.19d
SAH	1.92 ± 1.00c	1.86 ± 1.10bc	1.77 ± 0.88a	2.02 ± 1.11c	2.16 ± 1.14d	1.97 ± 0.96cd
MTC	1.71 ± 1.24b	1.88 ± 1.32bc	1.85 ± 1.16a	1.89 ± 1.37b	2.02 ± 1.51c	1.89 ± 1.24b
MTH	1.65 ± 1.00b	1.77 ± 1.06bc	1.84 ± 1.09a	1.88 ± 1.13b	1.88 ± 1.11b	1.85 ± 0.91b
ESC	1.92 ± 1.47c	2.01 ± 1.60c	2.27 ± 1.37b	2.14 ± 1.59c	2.24 ± 1.71d	1.90 ± 1.44bc
ESH	1.94 ± 1.26cd	1.90 ± 1.23bc	2.23 ± 1.35b	2.03 ± 1.30c	2.17 ± 1.35d	1.83 ± 1.08b
Friedman test	176.808	246.979	40.095	275.042	383.830	441.899
Asymptotic Sig.	*P* < 0.05	*P* < 0.05	*P* < 0.05	*P* < 0.05	*P* < 0.05	*P* < 0.05

Means in columns followed by different letters are different at the Bonferroni-adjusted significance level *P* ≤ 0.004 (Friedman test followed by Wilcoxon signed-rank post hoc tests with Bonferroni’s correction for multiple comparisons)

*BSC* body size–cock, *BSH* body size–hen, *SFC* supplementary feed consumption–cock, *SFH* supplementary feed consumption–hen, *ENH* egg number–hen, *EZH* egg size–hen, *SAC* scavenging ability–cock, *SAH* scavenging ability–hen, *MTC* meat taste–cock, *MTH* meat taste–hen, *ESC* ease of sales–cock, *ESH* ease of sales–hen, *SD* standard deviation

^a^The lower the mean, the more important the trait

Across genotypes, higher ratings of BSC and BSH were more (*P* ≤ 0.001) evident in FUNAAB Alpha, Sasso, Noiler, and Kuroiler (Table 8). However, ENH and EZH were more prioritized (*P* ≤ 0.001) in Shika Brown. SFC (Noiler and Fulani) and SFH (Noiler, Fulani and FUNAAB Alpha) were also highly rated. Preferences for SAC and SAH were higher (*P* ≤ 0.001) in Fulani, FUNAAB Alpha, and Shika Brown (also have higher rating for MTC and MTH). There was almost equal preference for ease of sales of cocks and hens.

**Table 8:** Mean ranks of traits preference across six chicken genotypes and their significance level according to Kruskall–Wallis test

	Genotype						
	Shika Brown	FUNAAB Alpha	Fulani	Kuroiler	Sasso	Noiler	
Traits	Mean rank	Mean rank	Mean rank	Mean rank	Mean rank	Mean rank	Kruskall–Wallis test
BSC	1082.90b	971.94a	1351.16c	1018.16ab	1010.99ab	1012.96ab	40.292**
BSH	1088.59b	974.47a	1230.45c	1023.56ab	1010.54a	1002.51a	21.008**
SFC	1013.94b	991.78b	975.38ab	1014.69b	1096.48c	911.83a	28.286**
SFH	1005.98b	971.39ab	939.06ab	1001.40b	1102.78c	910.31a	32.361**
ENH	801.83a	878.48b	1059.23c	982.83bc	1042.54c	925.90b	49.808**
EZH	821.92a	924.11b	1197.42d	936.70bc	1007.94c	951.92bc	39.894**
SAC	965.69ab	946.06a	885.13a	1033.12b	1068.86c	1021.86bc	16.406**
SAH	982.42ab	912.29a	883.56a	1013.05b	1077.72c	1016.14bc	22.065**
MTC	911.95a	1029.04b	1059.87b	1000.97b	1018.89b	1055.10b	14.402*
MTH	877.24a	970.45b	1012.48bc	1010.54bc	999.60bc	1056.98c	21.744**
ESC	938.38a	973.57a	1164.73b	1015.77ab	1016.11ab	957.03a	14.225*
ESH	933.71a	927.94a	1094.91b	982.25a	1028.14b	926.91a	16.222**

The lower the mean rank, the more important the trait. Means followed by different letters in rows are different [Kruskall–Wallis test followed by Mann–Whitney *U* tests (*P* ≤ 0.05)]

*BSC* body size–cock, *BSH* body size–hen, *SFC* supplementary feed consumption–cock, *SFH* supplementary feed consumption–hen, *ENH* egg number–hen, *EZH* egg size–hen, *SAC* scavenging ability–cock, *SAH* scavenging ability–hen, *MTC* meat taste–cock, *MTH* meat taste–hen, *ESC* ease of sales–cock, *ESH* ease of sales–hen

*, **Asymptotic significance at *P* ≤ 0.005 and *P* ≤ 0.001, respectively

Across all genotypes within a specific zone, traits preference varied significantly (*P* ≤ 0.01) (Table 9). In Kwara, farmers tended to favour BSC, BSH, ENH, SFH, EZH, SAH, and MTH. Farmers in Rivers ranked SAC and ESC lowest. In Imo, farmers were more favorably disposed to BSC, BSH, MTC, MTH, and EZH with less emphasis on SFC and SFH. In Nasarawa, SAH and SAC were ranked lowest while ESC, BSC, BSH, MTC, MTH, EZH, and ESH were highly ranked. BSC, BSH, and SAH were the traits prioritized in Kebbi.

**Table 9:** Mean (± SD) of traits preference in chicken and their significance level according to Friedman test within each zone

	Zone				
	Kwara	Rivers	Imo	Nasarawa	Kebbi
Traits	Mean^a^	Mean^a^	Mean^a^	Mean^a^	Mean^a^
BSC	1.60 ± 0.81a	1.68 ± 1.12a	1.54 ± 0.83a	1.34 ± 0.73ab	1.16 ± 0.53a
BSH	1.65 ± 0.83a	1.75 ± 1.12b	1.64 ± 0.93b	1.37 ± 0.72ab	1.22 ± 0.56b
SFC	2.52 ± 1.50b	2.20 ± 1.10c	2.31 ± 1.23e	1.69 ± 1.10ef	1.82 ± 1.23e
SFH	2.34 ± 1.24b	2.18 ± 1.06cd	2.27 ± 1.15e	1.72 ± 1.07f	1.75 ± 1.07de
ENH	2.32 ± 1.46b	2.06 ± 0.99c	2.03 ± 1.13d	1.52 ± 0.78de	1.65 ± 0.98cd
EZH	2.36 ± 1.48b	2.07 ± 1.06c	1.88 ± 1.02c	1.49 ± 0.76cd	1.78 ± 1.07de
SAC	2.79 ± 1.77d	2.34 ± 1.23d	2.11 ± 1.07d	1.89 ± 1.02g	1.66 ± 1.05cd
SAH	2.56 ± 1.44bc	2.17 ± 1.00c	1.98 ± 0.89cd	1.88 ± 1.03g	1.56 ± 0.75c
MTC	2.97 ± 2.01e	2.14 ± 1.52c	1.69 ± 1.01b	1.41 ± 0.61bc	1.66 ± 0.99cd
MTH	2.72 ± 1.54cd	1.91 ± 1.02bc	1.73 ± 0.93b	1.44 ± 0.62bcd	1.62 ± 0.70cd
ESC	3.29 ± 2.04f	2.36 ± 1.69d	1.90 ± 1.25cd	1.32 ± 0.68a	1.91 ± 1.52e
ESH	2.99 ± 1.64e	2.09 ± 1.13c	1.99 ± 1.20cd	1.49 ± 0.84cd	1.75 ± 1.06de
Friedman test (chi-square)	388.533	232.91	378.733	484.311	375.744
Asymptotic significance	*P* < 0.05	*P* < 0.05	*P* < 0.05	*P* < 0.05	*P* < 0.05

Means in columns followed by different letters are different at the Bonferroni-adjusted significance level *P* ≤ 0.004 (Friedman test followed by Wilcoxon signed-rank post hoc tests with Bonferroni’s correction for multiple comparisons)

*BSC* body size–cock, *BSH* body size–hen, *SFC* supplementary feed consumption–cock, *SFH* supplementary feed consumption–hen, *ENH* egg number–hen, *EZH* egg size–hen, *SAC* scavenging ability–cock, *SAH* scavenging ability–hen, *MTC* meat taste–cock, *MTH* meat taste–hen, *ESC* ease of sales–cock, *ESH* ease of sales–hen, *SD* standard deviation

^a^The lower the mean, the more important the trait

Trait preferences irrespective of chicken genotypes varied across the five zones (*P* ≤ 0.01) (Table 10). BSC, BSH, SAC, and SAH were ranked highest in Kebbi compared to others. However, farmers in Nasarawa attached more importance to EZH, MTC, MTH, ESC, and ESH in comparison with their counterparts from other zones. Farmers in Kwara had the least ranking for most of the traits. However, there was similarity in the ranking of the traits between farmers in Rivers and Imo.

**Table 10:** Mean ranks of traits preferred in the choice of chicken breeding stock across zones and their significance according to Kruskall–Wallis test

	Zone						
	Kwara	Rivers	Imo	Nasarawa	Kebbi		
Traits	Mean rank	Mean rank	Mean rank	Mean rank	Mean rank	Kruskall–Wallis test	Asymptotic significance
BSC	1189.20d	1101.00c	1148.59cd	934.50b	815.54a	160.429	≤ 0.01
BSH	1162.02c	1108.26c	1135.43c	919.35b	826.32a	137.414	≤ 0.01
SFC	1206.66c	1133.27b	1110.27b	777.76a	814.81a	217.365	≤ 0.01
SFH	1181.58b	1126.19b	1097.60b	801.50a	793.50a	199.583	≤ 0.01
ENH	1083.77b	1057.04b	998.20b	776.76a	817.72a	128.954	≤ 0.01
EZH	1101.44d	1024.71c	1036.81cd	763.92a	877.62b	108.628	≤ 0.01
SAC	1167.70c	1108.28c	1175.64c	916.90b	760.55a	161.247	≤ 0.01
SAH	1175.48d	1096.71c	1058.74c	932.34b	754.13a	154.146	≤ 0.01
MTC	1236.80d	1032.73c	1006.82bc	838.42a	939.67b	123.535	≤ 0.01
MTH	1220.33d	1015.01c	1018.57bc	809.39a	933.24b	126.289	≤ 0.01
ESC	1327.31d	1040.45c	965.41bc	711.50a	902.63b	281.623	≤ 0.01
ESH	1277.50d	1020.14c	975.56c	743.18a	865.73b	214.355	≤ 0.01

*BSC* body size–cock, *BSH* body size–hen, *SFC* supplementary feed consumption–cock, *SFH* supplementary feed consumption–hen, *ENH* egg number–hen, *EZH* egg size–hen, *SAC* scavenging ability–cock, *SAH* scavenging ability–hen, *MTC* meat taste–cock, *MTH* meat taste–hen, *ESC* ease of sales–cock, *ESH* ease of sales–hen

The lower the mean rank, the more important the trait. Means followed by different letters in rows are different [Kruskall-Wallis test followed by Mann–Whitney *U* tests (*P* ≤ 0.05)]

Within each gender, trait preference varied significantly (*P* ≤ 0.01) with the exception of BSC, BSH, MTC, and MTH that were highly ranked by both male and female farmers (Table 11). ENH, EZH, ESC, ESH, SAC, and SAH were more rated by the male farmers.

**Table 11:** Mean (± SD) of traits preferred by male and female chicken farmers according to Friedman test

	Gender	
	Female	Male
Traits	Mean^a^	Mean^a^
BSC	1.46 ± 0.86a	1.44 ± 0.82a
BSH	1.54 ± 0.90b	1.47 ± 0.82a
SFC	2.03 ± 1.21ef	2.18 ± 1.33d
SFH	2.00 ± 1.13e	2.11 ± 1.18d
ENH	1.92 ± 1.15de	1.80 ± 0.97b
EZH	1.90 ± 1.13cd	1.83 ± 1.04b
SAC	2.12 ± 1.27f	2.09 ± 1.25d
SAH	2.00 ± 1.08e	1.99 ± 1.03c
MTC	1.91 ± 1.38d	1.86 ± 1.28b
MTH	1.83 ± 1.06c	1.81 ± 1.01b
ESC	2.07 ± 1.57ef	2.05 ± 1.58c
ESH	1.99 ± 1.26de	2.00 ± 1.25c
Friedman test (chi-square)	821.347	504.187
Asymptotic significance	*P* < 0.05	*P* < 0.05

Means in columns followed by different letters are different at the Bonferroni-adjusted significance level *P* ≤ 0.004 (Friedman test followed by Wilcoxon signed-rank post hoc tests with Bonferroni’s correction for multiple comparisons)

*BSC* body size–cock, *BSH* body size–hen, *SFC* supplementary feed consumption–cock, *SFH* supplementary feed consumption–hen, *ENH* egg number–hen, *EZH* egg size–hen, *SAC* scavenging ability–cock, *SAH* scavenging ability–hen, *MTC* meat taste–cock, *MTH* meat taste–hen, *ESC* ease of sales–cock, *ESH* ease of sales–hen

^a^The lower the mean, the more important the trait

SFC (ranked higher by males) was the only trait significantly (*P* ≤ 0.05) influenced by gender (Table 12). However, the significance values of SFH (females; *P* ≤ 0.055) and BSH (males; *P* ≤ 0.082) were closer to (*P* ≤ 0.05) compared with those of ENH, ESC, BSC, SAC, EZH, MTC, ESH, and MTH, respectively.

**Table 12:** Mean ranks of traits preferred by male and female farmers in the choice of chicken breeding stock according to Kruskall–Wallis test

	Gender			
	Female	Male		
Traits	Mean rank	Mean rank	Kruskall–Wallis test	Asymptotic significance
BSC	1034.74	1018.15	0.511	0.475^ns^
BSH	1038.15	1034.74	3.020	0.082^ns^
SFC	987.27	1047.05	5.197	0.023*
SFH	983.35	1033.43	3.680	0.055^ns^
ENH	954.16	926.38	1.227	0.268^ns^
EZH	951.77	941.27	0.176	0.675^ns^
SAC	1016.57	1002.10	0.306	0.580^ns^
SAH	1005.76	1006.51	0.001	0.977^ns^
MTC	1013.95	1007.77	0.059	0.808^ns^
MTH	994.15	993.68	0.000	0.985^ns^
ESC	997.63	975.15	0.784	0.376^ns^
ESH	970.47	968.99	0.003	0.954^ns^

The lower the mean rank, the more important the trait

*BSC* body size–cock, *BSH* body size–hen, *SFC* supplementary feed consumption–cock, *SFH* supplementary feed consumption–hen, *ENH* egg number–hen, *EZH* egg size–hen, *SAC* scavenging ability–cock, *SAH* scavenging ability–hen, *MTC* meat taste–cock, *MTH* meat taste–hen, *ESC* ease of sales–cock, *ESH* ease of sales–hen

*Significant at *P* ≤ 0.05; ^ns^not significant

Supplementary feed consumption (0.90), scavenging ability (0.87), meat quality trait (0.86), ease of sales (0.85), body size (0.83), and egg trait (0.80) measurements were strongly and significantly (*P* ≤ 0.01) related (Table 13). The correlation coefficients between MTC and ESC (0.65) and MTH and ESH (0.68) were also high (*P* ≤ 0.01). The relationship between MTC and ESH (0.60) as well as that of MTH and ESC (0.62) was equally strong (*P* ≤ 0.01).

**Table 13:** Spearman’s rank order correlations of farmers’ traits of preference

Traits	BSC	BSH	SFC	SFH	ENH	EZH	SAC	SAH	MTC	MTH	ESC	ESH
BSC		0.83	0.35	0.33	0.41	0.44	0.25	0.27	0.33	0.33	0.44	0.44
BSH			0.30	0.31	0.38	0.43	0.22	0.25	0.30	0.32	0.39	0.43
SFC				0.90	0.30	0.27	0.50	0.50	0.39	0.39	0.40	0.42
SFH					0.31	0.28	0.50	0.52	0.38	0.38	0.38	0.41
ENH						0.80	0.32	0.36	0.46	0.45	0.47	0.46
EZH							0.31	0.34	0.45	0.46	0.49	0.49
SAC								0.87	0.44	0.42	0.38	0.37
SAH									0.46	0.47	0.39	0.42
MTC										0.86	0.65	0.60
MTH											0.62	0.68
ESC												0.85

Significant at *P* ≤ 0.01 for all correlation coefficients

*BSC* body size–cock, *BSH* body size–hen, *SFC* supplementary feed consumption–cock, *SFH* supplementary feed consumption–hen, *ENH* egg number–hen, *EZH* egg size–hen, *SAC* scavenging ability–cock, *SAH* scavenging ability–hen, *MTC* meat taste–cock, *MTH* meat taste–hen, *ESC* ease of sales–cock, *ESH* ease of sales–hen

Two PCs were extracted which explained 65.3% (Table 14) of the variability in the dataset. The first PC with Eigen value 5.421 contributed 45.2% to the total variance. It was characterized by supplementary feed consumption–cock; supplementary feed consumption–hen; egg number–hen; egg size–hen; scavenging ability–cock; scavenging ability–hen; meat taste–cock; meat taste–hen; ease of sales–cock and ease of sales–hen. However, body size in both cock and hen had high and positive loadings on the second PC with eigenvalue 2.416 and 20.1% contribution to the variance total. The total Cronbach’s alpha value of 0.952 was very high, which is an indication of the reliability of the PCA. Irrespective of gender and agro-ecological zone, the farmers can be grouped into two: Those that emphasize body size in both cock and hen and those that attach more importance to supplementary feed consumption–cock; supplementary feed consumption–hen; egg number–hen; egg size–hen; scavenging ability–cock; scavenging ability–hen; meat taste–cock; meat taste–hen; ease of sales–cock and ease of sales–hen.

**Table 14:** Description of farmers’ attributes of preference based on principal components

Trait	PC 1	PC 2
Body size–cock	0.229	1.545
Body size–hen	0.180	0.725
Supplementary feed consumption–cock	0.509	0.013
Supplementary feed consumption–hen	0.509	0.011
Egg number–hen	0.475	0.048
Egg size–hen	0.454	0.075
Scavenging ability–cock	0.533	0.004
Scavenging ability–hen	0.567	0.007
Meat taste–cock	0.644	0.014
Meat taste–hen	0.663	0.019
Ease of sales–cock	0.618	0.045
Ease of sales–hen	0.636	0.043
Eigenvalue	5.421	2.416
% of total variance	45.2	20.1
Cronbach’s alpha	0.893	0.556

## Discussion

The preponderance of females over males could be attributed to the fact that the primary targets of ACGG project are women and youth. This could have influenced the deliberate selection of more female households than their male counterparts. However, it is generally believed that more women are involved in poultry activities compared to men. This was corroborated by earlier studies (Bagnol 2009; Paudel et al. 2009; Fida et al. 2018).

The high preference for FUNAAB Alpha, Sasso, and Noiler birds in the present study could be due to their desirable performance in the field. This could have been influenced mainly by their body size and egg number. Although Kuroiler was ranked fourth, it was able to compete well with Sasso and Noiler chicken. This implies that in the case of non-availability of the latter, Kuroiler could be a good substitute. The low ranking of Shika Brown might be attributed to the fact that the breed was developed mainly for egg production unlike others that are dual-purpose. The least preference for Fulani chicken could be as a result of its low productivity compared to other genotypes [6-week body weight of 416.82 g (Sasso), 450.86 g (Kuroiler), and 228.66 g (Fulani) (Yakubu and Ari 2018); 20-week body weight (cocks) of 1.3 g (Fulani), 2.1 g (FUNAAB Alpha), 1.7 g (Shika Brown), 2.9 g (Kuroiler), 3.0 g (Sasso), and 2.6 g (Noiler) (Adebambo et al. (2018)]. However, this genotype is renowned for its high adaptability to the prevailing hot-dry tropical environment of Nigeria (Yakubu and Ari 2018) and good scavenging ability. Some of the merits indicated by farmers for the choice of a particular genotype in the current study are similar to the egg productivity, body size and fast growth traits reported by Sisay et al. (2018) and Mahoro et al. (2018). Gender differences in the present study as regards the choice of Sasso chicken breed may be attributed to poultry keeping objectives and varied importance attached to the chicken genotype by both male and female farmers.

Within each zone, traits of preference for selection of breeding stock in the present study tended towards body size, egg number, egg size, and meat taste. The observation on body size is in consonance with the findings of Muchadeyi et al. (2009) where the trait was ranked first among the criteria for choosing chicken breeding stock. Similarly, Mahoro et al. (2018) included body size and egg yield among the important economic traits to select the indigenous chickens. In a related study, Markos et al. (2016) ranked egg number and body weight as first and second, respectively, while Asmelash et al. (2018) reported that egg size was highly rated compared to other traits in village chicken. Meat quality in form of good taste is an important trait in the poultry industry. It has been recommended that breeding strategies should aim not only at the growth and performance of chicken, but also put into consideration the qualitative aspects of meat (Paiva et al. 2018).

The varying ranking of the traits of preference across zones in the present study could be attributed to heterogeneity in production environments. This was quite more evident between the sub-humid agro-ecological zones (Nasarawa and Kebbi) and their humid counterparts (Kwara, Rivers and Imo). However, the current findings are at variance with the submission of Markos et al. (2016) where there was no variability across agro-ecological zones in the ranking indices of chicken producers’ trait preferences.

The preference for body size within gender and the high ranking of egg number, egg size, meat taste, and ease of sales by male farmers in the current study might not be unconnected with their direct monetary values as consumers may be willing to pay premium with a unit increase in the traits. The easier the sales of the birds, the more the income also generated. However, across gender preference for supplementary feed consumption by female farmers might be due to the extra nutrients the birds will derive which may increase their production level. This is in consideration of the fact that women are predominantly involved in feeding chickens. This present information may inform breeding management decisions along gender mainstreaming in the study localities. In a related study in other species, Marshall et al. (2016) reported that gender differences may result from production objectives and the specific roles and responsibilities of males and females in traditional livestock rearing. This is linked to the constant state of change, evolution and development of traditional gender roles (Paudel et al. 2009; Karmebäck et al., 2015). However, the best way gender-differentiated trait preferences could make sense is to understand how such preferences reflect underlying gender differences in “assets, markets, information, and risk, and the ways institutions and policies condition these” (Ashby, 2018).

The strong positive relationship between supplementary feed consumption and scavenging ability is not quite unexpected since feed intake will increase correspondingly with increase in the ability to search for feed resources within the environment. In the same vein, an improvement in the taste of chicken meat may facilitate sales of the live chicken/chicken products. According to Northcutt (2009), a quality attribute determining poultry meat acceptability is flavor which might affect its subsequent sales (Kyarisiima et al. 2011). The relationships among the traits of preference in the present study permitted the possible grouping of the farmers along the line of preferred traits using PCA.

Two distinct groups of households keeping chickens in the sample population emerge, each displaying differing preferences for the chicken traits. This indicates the importance of considering heterogeneity within population segments as it provides a useful framework for adapting breeding policy interventions to specific producer segments. The present clustering could be attributed to individual differences in perceptions of trait of importance, the production objectives, social-cultural beliefs and livelihood strategies. Where resources are scarce, it is possible that genetic improvement of body size may meet the production objective of a particular group of farmers. On the other hand, there is another group which breeding objective emphasizes parameters such as supplementary feed consumption, egg number and size, scavenging ability, meat quality, and ease of sales. Such group may also be targeted during future poultry breeding and marketing interventions. PCA can be used for ranking and grouping (Ajayi et al. 2012; Lopes et al., 2013) and to explore the relationship between traits in a dataset (Pinto et al. 2006).

## Conclusion

The present study revealed equal ranking of FUNAAB Alpha, Sasso, and Noiler, followed by Kuroiler, Shika Brown, and Fulani chickens across five agro-ecological zones in Nigeria. More male farmers indicated preference for Sasso birds only across zones which could mainly be due to varying production objective. Traits of economic importance that appeared consistent in selecting breeding stock were body size, egg number, egg size, and meat taste. However, gender-differentiated trait preference was evident in supplementary feed consumption (female farmers) only. The chicken farmers were distinctly assigned into two groups (body size and non-body size traits) using categorical principal component analysis. These findings when combined with quantitative on-farm data have implications for future breeding programs geared towards increased chicken production and productivity in the tropics using bottom-top approach.
